# Post-marketing Study of Linagliptin: A Pilot Study

**DOI:** 10.3389/fphar.2019.00576

**Published:** 2019-05-24

**Authors:** Gabrielle Kéfrem Alves Gomes, Mariana Linhares Pereira, Cristina Sanches, André Oliveira Baldoni

**Affiliations:** Grupo de Pesquisa em Epidemiologia e Avaliação de Novas Tecnologias em Saúde, Universidade Federal de São João del-Rei, Divinópolis, Brazil

**Keywords:** linagliptin, Dipeptidyl peptidase 4 inhibitors, diabetes mellitus type 2, effectiveness, safety, pharmacovigilance, pharmacoepidemiology

## Abstract

**Introduction:**

Linagliptin is a high-cost oral antidiabetic that has been widely used, and studies on its effectiveness and safety for the treatment of type 2 diabetes mellitus (DM2) in the real world is rare and necessary.

**Objective:**

To analyze the values of glycated hemoglobin (HbA1c) and adverse events before and after the use of linagliptin in the post-marketing context of a pilot study.

**Methods:**

This is a descriptive observational and exploratory study with a retrospective longitudinal approach, conducted between January 2014 and December 2016. All patients who participated in the study were over 18 years of age, with DM2, assisted by the Brazilian Public Health System (*Sistema Único de Saúde* – SUS) and had been indicated for use of linagliptin. The users were followed up and the variables of interest were collected from a computerized health information system (*sistema informatizado de saúde* – SIS) and patient records. For effectiveness analysis, HbA1c before (T_0_) and after (T_1_) the use of linagliptin was considered in patients registered as having collected linagliptin at the pharmacy for at least three consecutive months. For safety analysis, registered adverse events (AE) were verified in patients’ records. The sample was stratified according to the pharmacotherapeutic scheme of the users. To compare the means before (T_0_) and after (T_1_), a paired *t*-test (data with normal distribution) and Wilcoxon Signed Rank Sum test (non-normal distribution data) were performed.

**Results:**

Considering the total population of the study, in a different pharmacotherapeutic regimen, a median reduction in HbA1c of -0.86% (*p* < 0.05) was observed. After stratification by pharmacotherapeutic regimen, the most significant reduction of HbA1c was -1.07% (*p* = 0.014) for the linagliptin group associated with insulins and oral antidiabetic agents (*n* = 13). On the other hand, patients taking linagliptin in monotherapy had the lowest HbA1c reduction, -0.48% (*p* > 0.05). AE occurred in 12 (36.4%) patients, and 16.7% were in monotherapy.

**Conclusion:**

Linagliptin did not presented, in real world, the desired performance as showed in randomized premarketing clinical trials and it should be carefully evaluated in public health services.

## Introduction

Diabetes mellitus type 2 (DM2) is a chronic disease highly prevalent in the adult population. The main objectives of DM2 treatment are metabolic control, the reduction of microvascular and macrovascular complications associated with the disease, as well as the reduction of its acute manifestations. To meet these goals it is necessary that blood glucose reach normal levels, both in fasting and in the postprandial period. Regarding the choice of pharmacological therapy, this should take into consideration the mechanisms of insulin resistance, secretory capacity of the pancreas, metabolic disorders involved, and the complications of DM2 present (American Diabetes Association [ADA], 2018). In healthy individuals, glucagon-like peptide 1 (GLP-1) and glucose-dependent insulinotropic polypeptide (GIP), which are intestinal hormones or incretins, account for up to 70% of the insulin response, as they contribute to the modulation of pancreatic beta-cell activity, stimulating insulin secretion (Morris et al., 2013; American Diabetes Association [ADA], 2018).

In DM2 there is a reduced response of the insulin effect, which alters the regulation of the amount of glucose present in the blood, contributing to a lack of glycemic control of the sick individual. In this sense, incretin analogs and inhibitors of the enzyme Dipeptidyl peptidase-4 (DPP-4) have been developed in order to potentiate the function of these endogenous hormones. Incretin-based therapy has been increasingly prominent among treatment options for type 2 diabetes (DM2) (Websky et al., 2013). Studies demonstrate the efficacy of these substances in glycemic control as well as in the weight reduction of these patients (International Diabetes Federation [IDF], 2017; American Diabetes Association [ADA], 2018).

As members of the incretin class, gliptins are the inhibitors of the DPP-4 enzyme. Linagliptin, a representative of this class, has a peculiar pharmacological profile: pharmacokinetics allowing only one daily administration and no dose adjustment requirement for patients with renal and hepatic dysfunction (Richard, 2014; American Diabetes Association [ADA], 2018). Linagliptin can be used both in monotherapy and in combination with other antidiabetic agents (Chen et al., 2015; Marx et al., 2015; Mikhael, 2016; Thrasher, 2016). Studies have demonstrated that this association of other antidiabetic agents with linagliptin has been shown to be effective and widely used in clinical practice in order to optimize treatment of DM2 (Defronzo et al., 2015; Haak, 2015).

Several clinical trials have shown that incretins, such as linagliptin, have been considered a great therapeutic promise in terms of effectiveness and safety (Chen et al., 2015; Defronzo et al., 2015; Haak, 2015; Marx et al., 2015; Mikhael, 2016; Thrasher, 2016). However, data on the use of this class in the real world in monotherapy or in combination are scarce (Barnett et al., 2013; Sortino et al., 2013; Richard, 2014). In addition, linagliptin, and other representatives of the class of gliptins, are on the list of medications to be avoided, according to data published in the journal “*Prescrire*” and there is concern about an unfavorable profile of adverse effects including urinary tract infections and upper respiratory tract infections (Prescrire International, 2017).

In addition, studies on the effectiveness and adverse event profile of linagliptin in the post-marketing context is rare and necessary. In this context, this study, considered a pilot, aims to reduce this gap between the use of linagliptin by patients in the “real world” and the evidence from randomized clinical trials in developed countries. This study aims is to analyze the values of glycated hemoglobin and adverse events before and after the use of linagliptin in the post-marketing context of a pilot study.

## Materials and Methods

### Study Design

This is a descriptive observational and exploratory study with a retrospective longitudinal approach (Elseviers et al., 2016). The study was outlined and described following the recommendations of Kempen (2011).

### Setting

The city where the study was conducted has 21,3016 inhabitants, Human Development Index (HDI) of 0.764, and 43 primary health care units, and only one center of endocrinology.

### Participants

All patients served at health units of the Brazilian Public Health System (SUS) of the city of Divinópolis, in the state of Minas Gerais (MG) who received a medical indication for the use of linagliptin during the period from January 2014 to December 2016 were identified and considered eligible for the study. Identification was made through a computerized health information system (SIS) that records the medication dispensed to the patients.

All participants who took linagliptin for at least three consecutive months were considered. The 3-month period was established so that it was possible to analyze the effectiveness of linagliptin according to the time required for variation of HbA1c levels (Malta et al., 2010). Participants in concomitant use of other medicinal products of the DPP-4 inhibitor class were excluded. This information was collected together with the patient’s medical record and dispensing record.

### Variables and Data Source

The outcome variables considered were the effectiveness and safety of linagliptin. The analysis of the effectiveness of linagliptin was performed by comparing patients’ HbA1c values shortly before linagliptin (T_0_) and after the first 3 months of consecutive use of the medication (T_1_).

Safety was analyzed from the active search for adverse events (AE) registered in patients’ medical records during the period of linagliptin use. A list of adverse events related to linagliptin was developed to direct and systematize the search for AE reported in patients’ records. This list was constructed after a systematized search in the literature on adverse medication events (Andriolo and Vieira, 2005; Food And Drug Administration [FDA], 2013; ANVISA, 2017; Gomes et al., 2018). To investigate possible laboratory abnormalities, the results of microalbuminuria, urea, aspartate transaminase (AST), alanine aminotransferase (ALT), and gamma-glutamyltransferase (GAMA-GT) were investigated. Only the AE that were recorded in the patients’ records during the period of linagliptin use were considered for this analysis.

In addition to the outcome variables, the following variables were analyzed: (I) demographic data: gender, self-reported race; (II) clinical data: pharmacotherapy used for DM2, family history, presence of comorbidities, and time of diagnosis of DM2; (III) biochemical data: fasting glycemia, glycated hemoglobin, postprandial glucose, creatinine, and urea. Medical records were used to define the presence of alcoholism, degree of obesity, renal failure, and other diagnoses.

### Statistics

Data analysis was performed with STATA software – Data Analysis and Statistical Software, version 12.0. To compare the biochemical tests before (T_0_) and after (T_1_) the use of linagliptin, the normality of the data of each variable was analyzed through the value of skewness and kurtosis, after which comparative analyses were performed between groups. For the data with normal distribution the paired *t*-test was performed and for the data with non-normal distribution the Wilcoxon Signed Rank Sum test was used. For the variable DM2 diagnosis time, the data were classified into groups according to the interquartile ranges observed (<25%, between 25 and 50%, >50%). To analyses differences in the values of HbA1c before (T_0_) and after (T_1_) the treatment with linagliptin, and stratification by pharmacotherapy of the medications of the patients was used paired *t*-test. All analyses were performed considering the level of significance of 5% and confidence level of 95%.

### Ethics Statement

This research was approved by Ethics in Research Committee of the Federal University of São João del-Rei (UFSJ), whose approval protocol is 1,827,849.

## Results

It was observed that 108 participants had access to linagliptin for at least 1 month, however, only 33 (30.6%) had access for at least three consecutive months (inclusion criteria of the study). Table 1 shows the profile of the 33 patients. It was observed that the majority of the patients were female (72.7%), evenly distributed among the age groups. The majority of patients were mixed race (36.3%), non-alcoholic (75.8%) and non-smokers (72.7%), and 48.5% reported a sedentary lifestyle. Regarding baseline glycated hemoglobin values (T_0_), it was observed that the majority of the patients (63.7%) had values above 9%. However, a significant number of patients (33%) presented HbA1c values within the normal range before starting treatment.

**Table 1:** Sociodemographic characteristics; lifestyle and glycated hemoglobin (HbA1c) in patients with linagliptin use in the period 2014–2016 (*n* = 33).

Variable	*n* (%)
**Gender**	
Female	24 (72.7)
Male	9 (27.3)
**Age range (years)**	
30–49	6 (18.1)
50–59	9 (27.3)
60–69	9 (27.3)
Over 70	9 (27.3)
**Self-reported race**	
Black	2 (6.1)
Mixed	12 (36.3)
White	7 (21.2)
Oriental	2 (6.1)
Not informed	10 (30.3)
**Alcoholism**	
Yes	2 (6.1)
No	25 (75.8)
Not informed	6 (18.1)
**Smoker**	
Yes	2 (6.1)
No	24 (72.7)
Not informed	7 (21.2)
**Sedentary**	
Yes	16 (48.5)
No	11 (33.3)
Not informed	6 (18.2)
**Range of HbA1c values prior to linagliptin use**	
<6%	11 (33.3)
6–8%	1 (3.0)
>9%	21 (63.7)


*Data collected at T_*0*_ – prior to the use of linagliptin.*

About the clinical characteristics of the patients, concerning the time of diagnosis for DM2, a higher prevalence of diagnostic times of 7–15 years (48.5%) was observed in this population. Regarding the observed comorbidities, 78.8% of the patients had systemic arterial hypertension, 36.4% dyslipidemia, and 27.3% cardiovascular disease. As for family history, the most prevalent diseases were cardiovascular disease (30.3%), diabetes mellitus (21.2%), and systemic arterial hypertension (15.2%) (Table 2).

**Table 2:** Clinical characteristics of patients in continuous use of linagliptin attended by the Brazilian Public Health System (SUS) from 2014 to 2016 (*n* = 33).

Observed characteristics	*n* (%)
**Time of diagnosis in years (*n* = 29)**	
Less than 7	8 (24.2)
From 7 to 15	16 (48.5)
More than 15	6 (27.3)
**Comorbidities (*n* = 31)**	
Systemic arterial hypertension	26 (78.8)
Dyslipidemia	12 (36.4)
Cardio vascular disease^1^	10 (27.3)
Obesity^2^	8 (24.2)
Chronic kidney disease^3^	7 (21.2)
Hypothyroidism	6 (18.2)
Depression	5 (15.2)
Cataract	2 (6.1)
Fibromyalgia	2 (6.1)
**Family history (*n* = 29)**	
Cardiovascular disease	10 (30.3)
Diabetes mellitus (unspecified)	7 (21.2)
Systemic arterial hypertension	5 (15.2)
Hypothyroidism	2 (6.7)
Others^4^	4 (12.2)


*Data collected at T_*0*_ – prior to the use of linagliptin. ^*1*^Cardiovascular diseases considered for this analysis were: cerebrovascular accident (CVA), congestive heart failure (CHF), unstable angina. ^*2*^Patients with degrees of obesity type I and type II were grouped in this class of clinical condition. ^*3*^Patients with degrees of renal failure III and III were grouped in this class of clinical condition. ^*4*^Other family histories found less frequently: throat cancer, bowel cancer, breast cancer, and hearing loss.*

Regarding the laboratory parameters, there was no statistical difference before and after the use of linagliptin (T_0_ and T_1_) (Table 3). In the results of microalbuminuria, AST, ALT, and GAMA-GT, which were investigated to analyze the safety associated with the use of linagliptin, no altered values were observed. However, it was not possible to carry out the statistical analyses due to the scarce recording of these data.

**Table 3:** Comparison of laboratory parameters before (T_0_) and after (T_1_) the continued use of linagliptin by patients attended by the Brazilian Public Health System (SUS) from 2014 to 2016 (*n* = 33).

Laboratory parameter	Reference value	Before (T_0_)^∗^	After (T_1_)^∗^	*p*-value^∗^
Fasting glycemia (*n* = 32)	<130 mg/dL	171.8 (114-190)	139.4 (101.5-156.5)	0.1299
Postprandial glucose (*n* = 23)	<180 mg/dL	205.7 (143-252)	189.3 (120-237)	0.7320
Serum creatinine (*n* = 28)	From 0.4 to 1.3 mg/dL	1.4 (0.93-1.48)	1.1 (0.9-1.3)	0.7208
Serum urea (*n* = 25)	From 10 to 45 mg/dL	49.1 (26-63)	47 (27-62)	0.9256


*^∗^Non-parametric data presented in median (interquartile range: 25–75%) and statistical analyzes performed by the Wilcoxon Signed Rank Sum Test. Source: ADA, 2018 and VII Brazilian Guidelines on Hypertension. HbA1c, glycated hemoglobin.*

In relation to pharmacotherapy, the association of “linagliptin with other oral antidiabetics and insulin” was the most used pharmacotherapeutic scheme among patients (45.4%). Data observed at baseline showed patients with microvascular complications such as chronic kidney disease (21.2%), diabetic retinopathy (12.1%), diabetic neuropathy (6.1%), amputation (3.1%), and glaucoma (3.1%).

Regarding the effectiveness of linagliptin, it was observed that the mean HbA1c of the patients reduced from 8.94% (±2.2) to 8.08% (±1.7). These data correspond to an absolute reduction of -0.86% (*p* < 0.05) in HbA1c values. After stratification of the sample according to the pharmacotherapeutic scheme for DM2 used by the patients, the reduction of HbA1c was lower when linagliptin was used as monotherapy (Table 4).

**Table 4:** Differences in the values of HbA1c before (T_0_) and after (T_1_) the treatment with linagliptin, and stratification by pharmacotherapy of the medications of the patients served by the Brazilian Public Health System (SUS) from 2014 to 2016 (*n* = 33).

Pharmacotherapeutic scheme	% HbA1c T_0_ (DP)^∗^	% HbA1c T_1_ (DP)^∗^	Effectiveness	*p*-value of	Frequency of
			(HbA1c: T_1_–T_0_)	effectiveness	adverse events (%)
Linagliptin (*n* = 6)	8.62 (1.3)	8.14 (1.5)	-0.48	0.177	16.70
Linagliptin + oral antidiabetics (*n* = 11)	7.80 (1.3)	7.36 (1.0)	-0.44	0.15	16.70
Linagliptin + insulins (*n* = 3)	11.53 (4.4)	9.23 (2.8)	-2.3	0.095	8.30
Linagliptin + insulin + oral antidiabetics (*n* = 13)	9.47 (2.0)	8.40 (1.9)	-1.07	0.014*	58.30
All patients (*n* = 33)	8.94 (2.2)	8.08 (1.7)	-0.86	0.001*	36.40


*^∗^Parametric data presented on average (standard deviation) and statistical analyses performed by the paired *t*-test. HbA1c, glycated hemoglobin. Pharmacotherapeutic groups: LINA, linagliptin monotherapy; LINA + AO, linagliptin associated with oral antidiabetics; LINA + INS, linagliptin associated with insulins; LINA + INS + AO, linagliptin associated with insulin and oral antidiabetic agents.*

Among the 12 (36.4%) patients who presented AE records during the use of linagliptin, 16.7% were on monotherapy with linagliptin and 83.3% in association with other antidiabetics. Occurrences of 25 types of AE were observed and hypoglycemia corresponded to 20.0% of the total; 60.0% of the complaints about hypoglycemia occurred in the association of “linagliptin with insulin and other oral antidiabetics” (Table 5).

**Table 5:** Adverse events using linagliptin described in the records of patients served by the Brazilian Public Health System (SUS), from 2014 to 2016 (*n* = 33).

Profile of recorded adverse events (AE)	*n* (%)
Total number of patients with adverse events	12 (36.4%)
Number of registered AE	25
**Types of adverse events**	
Hypoglycemia^1^	5 (20.0)
Muscular pain^2^	3 (12.5)
Gastrointestinal^3^	3 (12.5)
Others^4^	14 (56.0)


*^*1*^Unspecified hypoglycemia (4); hypoglycemia at night (1). ^*2*^Pain in lower limbs (2); lower back pain (1). ^*3*^Gastrointestinal events: intestinal constipation (1); vomiting (1); diarrhea (1). ^*4*^Other: hepatomegaly, polydipsia, polyuria, polyphagia, weight loss, nocturia, altered sleep-wake cycle, edema, fever, fetid urine, weight gain, decreased visual acuity, dizziness, and otitis.*

## Discussion

In the effectiveness analysis of linagliptin in the present study, a difference of -0.86% (*p* < 0.05) of the mean values of HbA1c was observed, when considering the total population in use of the medication. However, when analyzing the effectiveness of linagliptin in monotherapy, the difference in HbA1c values was -0.48% (*p* > 0.05). Results of a phase III study that demonstrated its efficacy in monotherapy are close to the results here presented, with a difference in HbA1c of -0.67% after 24 weeks of study (Nogueira et al., 2014) and -0.87% after 12 weeks of study (Tang et al., 2015). In addition, according to the consensus algorithm for initiation and adjustment of therapy for DM2, the expected difference in HbA1c with iDPP-4 in monotherapy is -0.50 to -0.80%. It is also worth noting that treatment with this class has a neutral effect on weight, has long-term safety, but is expensive pharmacotherapy (Kawamori et al., 2012).

Therefore, it is important to note that this pilot study has limitations that limit the generalization of results, such as, the study has a small sample size and it is not a randomized clinical trial with control group. In addition, it was not possible to control confounders. Another point concerns the follow-up time of patients taking linagliptin, which was relatively short, so that it was not possible to observe probable AE associated with the chronic use of the medication. Also, it is important to note that it was not possible to evaluate adherence to treatment by primary and direct methods. This study also presents limitations inherent in observational studies, such as the lack of control of the researcher on the scenario investigated. In addition, information biases can be attributed to data collection performed with secondary sources of information.

The study by Nathan et al. (2009) and Lauand et al. (2014) suggests that the effect of linagliptin on the reduction of HbA1c appears to be moderate when compared to other oral agents such as metformin and sulphonylurea, reducing from 1.0 to 2.0%, and thiazolidinedione of 0.5–1.4%. The authors consider that linagliptin has a relatively low occurrence of hypoglycemia. Therefore, this medication has been proposed to be used as a second line therapy associated with metformin in the treatment of adult patients with DM2, or even as a first line therapy in those patients intolerant to metformin. The study also points to linagliptin as an option to be used in a triple pharmacotherapeutic scheme, as observed in this investigation, which would be in combination with oral antidiabetics and insulin.

Regarding the pharmacotherapy for DM2 used by patients included in the effectiveness analysis of the present study, it was found that linagliptin was indicated as monotherapy or associated with other antidiabetics and insulins. Regarding the treatment of DM2, in the current protocols there is no specific and clear information about which stage of the disease linagliptin is indicated (American Diabetes Association [ADA], 2018). However, there are premarketing studies that demonstrate the efficacy and safety of associating linagliptin with insulin receptor sensitizers, such as biguanides and glitazones (Haak, 2015) or with other medicinal products that act to stimulate insulin production and secretion (Ross et al., 2016) and also with insulin (Haak et al., 2013; Lauand et al., 2014; Defronzo et al., 2015).

Among the four pharmacotherapeutic groups used in conjunction with linagliptin, the insulin group in combination with oral antidiabetics was the most used among patients (45.4%). Although linagliptin was not approved in Brazil by the National Agency of Sanitary Surveillance (ANVISA) for use with insulin, an off-label use of this medication was observed in this study. However, linagliptin-specific warnings and precautions given by the Food and Drug Administration (FDA) indicate that when this medication is being used with an insulin secretagogue (e.g., sulphonylurea) or insulin, we should consider reducing the dose of the insulin or insulin secretagogue to reduce the risk of hypoglycemia. Despite the divergences of indication for the association of linagliptin and insulins between regulatory agencies, phase III studies demonstrate that the association of basal insulin and other DPP-4 inhibitors significantly improves glycemic control over placebo (Rosenstock et al., 2009; Barnett et al., 2013; Yki-Järvinen et al., 2013; Marra et al., 2017).

However, in spite of the investigations demonstrating efficacy and safety in the use of linagliptin associated with insulin, in none of them was justified the rationale of this association, since the progression of DM2 reflects in the reduction of the production of insulin by the organism, a consequence of the reduction of the functioning beta cells (International Diabetes Federation [IDF], 2017). Another important factor is that the studies do not define the time of diagnosis of the patients included, or an evaluation of the tests that prove the secretory capacity of the pancreas. In the study by Yki-Järvinen et al. (2013) and Lauand et al. (2014) they observed that the type of insulin used, basal or bolus, did not interfere with the efficacy and safety of the combination with linagliptin. The literature suggests that the use of iDDP-4 should be a co-adjuvant in the treatment of DM2 (Vilsbøl et al., 2010), but it is not yet clear what are the therapeutic regimens in which it is most effective.

Studies have shown the efficacy of linagliptin associated with other pharmacotherapeutic regimens such as with metformin, suggesting a 2.72% HbA1c difference (Haak, 2015). In the results found in this study, a difference of HbA1c of -0.44% (*p* = 0.150) was observed for the association of linagliptin and oral antidiabetics, which included metformin 850 mg, metformin XR 500 mg, glibenclamide 5 mg, and gliclazide 30 mg. The differences in the values found may be related to two main factors at T_0_ of the study, being (a) the difference of clinical parameters (diagnosis time, comorbidities, etc.), and (b) mean HbA1c. In the study by Ross et al., HbA1c at T_0_ was 9.80 (1.1)%, being higher than in this study’s population, which was 8.94 (2.2)%. According to the ADA, in patients with HbA1c values greater than 9%, gliptins may be more effective (American Diabetes Association [ADA], 2018). A meta-analysis involving 98 observational studies with 24,163 patients using iDPP-4 in different associations, attributed the cause of HbA1c reduction of 36.0% at the baseline level of HbA1c in patients. The study also found that variables such as prior oral treatment, age, gender, and body mass index (BMI), and the treatment time of the participants had no significant additional effect on the HbA1c reduction variance (Esposito et al., 2014b).

In the present study, a greater prevalence of diagnostic times of 7–15 years was observed, suggesting a population with a reduced secretory capacity of insulin by beta cells of the pancreas. In the analysis between the time of diagnosis of DM2 and the reduction in HbA1c values it was not possible to establish a correlation between the two variables. Even if these variables did correlate, it is admitted that this is a heterogeneous population with different pharmacotherapeutic regimens associated with linagliptin. Therefore, the reduction of observed HbA1c could not be attributed in a restricted way to the effectiveness of linagliptin, since insulin behaves as a powerful agent for the reduction of glucose (Esposito et al., 2015).

The literature indicates that the HbA1c reduction profile of iDPP-4 reduces with the treatment time, showing greater effectiveness in the first weeks (Vilsbøl et al., 2010). However, the time of accomplishment of the present study did not allow for the observation of this effectiveness profile, which suggests the importance of additional investigations with longer follow-up times.

Regarding the safety results of linagliptin, it was observed that more than one third of the 33 patients who used the medication continuously had some adverse event described in the literature related to linagliptin. The pharmacotherapeutic regimen that presented the most adverse events (58.3%) was that of triple pharmacotherapy (linagliptin + oral antidiabetic + insulin), with hypoglycemia being the most reported AE in this group. In this sense, considering the pharmacodynamics of these medications, it is valid to consider that this event may be related more strictly to the use of insulin than to linagliptin. However, the study design and the co-medications used do not allow to infer the causality of the AE. In addition, information on the insulin doses used was not available. It is important to note that in this study only those AE that occurred after starting treatment with linagliptin were considered.

The total frequency of hypoglycemia in the 33 patients was approximately 15.0%. In the study by Gomis et al. (2012) and Esposito et al. (2014a), a similar frequency of hypoglycemia of 14.6% was observed in patients using linagliptin with other antidiabetics over a period of 24 weeks, twice the time of the present study (Esposito et al., 2014a).

In monotherapy with linagliptin the observed frequency of hypoglycemia was 16.7%, being a higher frequency when compared to studies by Haak et al. (2013), Defronzo et al. (2015), and Ross et al. (2016) whose incidence of this adverse event was lower than 8.0%. The present study differs from the clinical trials regarding the follow-up time of the participants, which ranged from 24 to 52 weeks in these studies, and also the characteristics of the population, since the clinical trials were controlled and since they excluded from the study any participants presenting comorbidities and who were inserted into the real world of polypharmacy.

Gomis et al. (2012) and Inagaki et al. (2013) evaluated hypoglycemia in the linagliptin-associated groups of biguanide, glinid, glitazone, sulfonylurea, and α-glucosidase inhibitors. In that study, only in the groups treated with linagliptin associated with sulphonylurea did hypoglycemia occur (9.5 and 5.9%). The incidence of hypoglycemia was significantly lower (<4.0%) in other studies using this AE as one of the outcomes (Kawamori et al., 2012; Inagaki et al., 2013; Tang et al., 2015).

The literature reports that hypoglycemic events are rare because of the glucose-ingestion dependent action (Haak et al., 2013), but they occur predominantly when a DPP-4 inhibitor is associated with sulfonylureas (American Diabetes Association [ADA], 2018). In the present study six patients (18.2%) were using glibenclamide or gliclazide, which are representatives of sulfonylureas associated with hypoglycemia.

It is important to note that in most premarketing studies, patients with these clinical conditions were not eligible for the study because of exclusion criteria, or family history data were not assessed (Haak et al., 2013; Inagaki et al., 2013; Lauand et al., 2014; Defronzo et al., 2015; Tang et al., 2015). In view of this, the importance of the post-marketing studies that accompany, record, and analyze data on the use of the medication in the real world stands out. All these factors can justify the differences found in this study, both in the effectiveness results and those related to medication safety.

Although the laboratory parameters analyzed did not present statistically significant differences between T_1_ and T_0_, a reduction was observed in fasting glucose, postprandial glucose, serum creatinine and serum urea levels. The fact that these parameters did not show significant improvement in their values can be explained by the small sample size and the short follow-up period. In addition, it is important to note that the scarcity of recording in the patient’s medical record of safety parameters such as microalbuminuria, AST, ALT, and GAMA-GT suggests absence of clinical monitoring or non-occurrence of an adverse event.

A systematic review by Gomes et al. (2018) presented results from 16 randomized clinical trials, evaluating the effectiveness and safety of linagliptin. The study identified that 93.8% of the studies were funded by the pharmaceutical industry, which evidences the need for studies free of conflicts of interest (Andriolo and Vieira, 2005).

Regarding the strengths of the study, it should be considered that this is the first real-world investigation conducted with Brazilian patients who used linagliptin, free from the influence of the pharmaceutical industry, in which the pharmacotherapy studied is immersed in a complex scenario which is related to the existing comorbidities and the presence of other factors extrinsic to the participants. In this sense, it is valid to consider that 93.8% of the studies evaluating the safety of linagliptin are financed by the pharmaceutical industry, and most of them had a comparison with placebo rather than with conventional pharmacotherapies (Andriolo and Vieira, 2005). On the other hand, the results of this study cannot be generalized, given the small sample size and the specificity of the participants.

In summary, the relevance of post-marketing studies as a tool for decision makers is recognized, especially in the face of unfavorable economic scenarios. Pharmacoeconomic studies and with a greater number of patients are needed to subsidize information for more assertive choices, maximizing the benefits of investments, without compromising the sustainability of the public health system.

## Conclusion

In the real world, linagliptin presented lower performance than in randomized premarketing clinical trials. These results reinforce the relevance of post-marketing studies as a tool for decision, especially in the face of unfavorable economic scenarios. In addition, it is important that further research be conducted through pragmatic clinical trials to be performed to assess possible confounding variables of real-world, such as adherence and access to medications. Because in public health system is not feasible that the therapeutic alternative has only efficacy. It needs to be effective and efficient.

## Ethics Statement

This research was approved by Research Ethics Committee (CEP) involving Human Subjects of Federal University of São João del-Rei (UFSJ), Central-West Campus (CCO), whose approval protocol is 1,827,849.

## Author Contributions

GG, MP, CS, and AB contributed to conception and design of the study. GG organized the database and performed the statistical analysis. MP, CS, and AB wrote the first draft of the manuscript. All authors contributed to manuscript revision, and read and approved the submitted version.

## Conflict of Interest Statement

The authors declare that the research was conducted in the absence of any commercial or financial relationships that could be construed as a potential conflict of interest.
